# Macroecological patterns of American Cutaneous Leishmaniasis transmission across the health areas of Panamá (1980–2012)

**DOI:** 10.1016/j.parepi.2016.03.003

**Published:** 2016-03-18

**Authors:** Koji Yamada, Anayansi Valderrama, Nicole Gottdenker, Lizbeth Cerezo, Noboru Minakawa, Azael Saldaña, José E. Calzada, Luis Fernando Chaves

**Affiliations:** a Institute of Tropical Medicine (NEKKEN), Nagasaki University, Sakamoto 1-12-4, 852-8523 Nagasaki, Japan; b Graduate School of Biomedical Sciences, Nagasaki University, Sakamoto 1-12-4, 852-8523 Nagasaki, Japan; c Instituto Conmemorativo Gorgas de Estudios de la Salud (ICGES), Apartado Postal No. 0816-02593, Ciudad de Panamá, Panama; d Department of Veterinary Pathology, College of Veterinary Medicine, University of Georgia, Athens, GA 30602, USA; e Departamento de Epidemiología, Ministerio de Salud, Ciudad de Panamá, Panama; f Programa de Investigación en Enfermedades Tropicales (PIET), Escuela de Medicina Veterinaria, Universidad Nacional, Apartado Postal 304-3000, Heredia, Costa Rica

**Keywords:** El Niño, Reservoirs, Deforestation, Marginalization, Sand fly vectors

## Abstract

American Cutaneous Leishmaniasis (ACL) is a neglected vector-borne zoonosis that persists despite increasing socio-economic development and urbanization in Panamá. Here, we investigate the association between environmental changes and spatio-temporal ACL transmission in the Republic of Panamá (1980–2012). We employ a macroecological approach, where patterns of variation in ACL incidence at the spatially coarse-grained scale of health areas are studied considering factors linked to the ecology of ACL transmission. We specifically study impacts of climatic variability, measured by the different phases of El Niño Southern Oscillation (ENSO), within diverse ecosystems and sand fly (Diptera: Psychodidae) vector species, as well as heterogeneous local climatic patterns, deforestation, population growth rates, and changes in social marginalization. We found that over the study period, patterns of ACL incidence: (i) were asynchronous with clusters changing from east to west of the Panamá Canal, (ii) trends increased in the west, and decreased or remained nearly constant in the east, independent of human population growth, (iii) generally increased in years following El Niño, and (iv) decreased as forest cover increased. We found no significant association between changes in socio-economic indicators and ACL transmission. Regarding vector abundance and presence, we found that studies had been biased to locations east of the Panamá canal, and that, in general, the abundance of dominant vector species decreased during the cold phase of ENSO. Finally, our results indicate that a macroecological approach is useful to understand heterogeneities related to environmental change impacts on ACL transmission.

## 1 Introduction

American Cutaneous Leishmaniasis (ACL) is a neglected vector-borne disease, closely associated with environmental change and poverty (Alvar et al., 2006, Wijeyaratne et al., 1994). Annually, around 66,000 new ACL cases are reported in the New World, and 2188 ACL cases occur annually in Panamá (Alvar et al., 2012). The reported cases are believed to account for only 1/3 to 1/2 of the real number of cases (Alvar et al., 2012, Christensen et al., 1999). In Panamá, ACL is mainly endemic and enzootic (Christensen et al., 1983, Christensen et al., 1999, Miranda et al., 2009). The main ACL parasite is *Leishmania panamensis*. Nevertheless, a few cases due to other *Leishmania* spp. were reported a few decades ago (Christensen et al., 1983, Christensen et al., 1999, Miranda et al., 2009, Grimaldi et al., 1989, Kreutzer et al., 1991). Vectors include several *Lutzomyia* spp., commonly known as sand flies (Diptera: Psychodidae), and as “chitras” in the Republic of Panamá. *Lutzomyia gomezi*, *Lu. panamensis*, and *Lu. trapidoi*, are the dominant vector species in Panamá (Christensen et al., 1983, Calzada et al., 2013, Chaves et al., 2013, Dutari and Loaiza, 2014). Proven enzootic mammal reservoirs include *Choloepus hoffmanni* (two-toed sloth), and several rodents, mainly rice (*Oryzomys* spp.) and spiny (*Proechimys* spp.) rats (Herrer and Telford, 1969, Herrer et al., 1971, Telford et al., 1972, Herrer and Christensen, 1976). Control for this disease in Panamá is currently focused on free clinical treatment for laboratory diagnosed cases, and relatively little attention has been given to strategies focused on vector control or that exploit the understanding of ACL transmission eco-epidemiology (Chaves et al., 2013, Saldaña et al., 2013). However, recently, the number of ACL cases has been increasing in the Republic of Panamá (Saldaña et al., 2013), and renewed efforts to understand ACL eco-epidemiology and to improve ACL vector control and ACL diagnostics have been carried out in Panamá (Calzada et al., 2013, Chaves et al., 2013, Saldaña et al., 2013, Miranda et al., 2012). Moreover, the increasing trend in ACL incidence contradicts previous proposals that socio-economic development and urbanization would eliminate the disease (Herrer and Christensen, 1976, Christensen and de Vasquez, 1982).

It is unclear what has been driving ACL incidence changes in Panamá. Competing hypotheses for the recent increase in ACL incidence include: (1) shifts of reservoir hosts from wildlife (Herrer and Telford, 1969, Herrer et al., 1971, Herrer et al., 1973, Telford et al., 1972) to domestic mammals (Calzada et al., 2015a, Travi et al., 2006, Chaves et al., 2007); (2) changes in vector species composition (Calzada et al., 2013, Valderrama et al., 2008, Valderrama et al., 2011); (3) human population increase in endemic areas (Alvar et al., 2012, Chaves et al., 2008a), and (4) transmission exacerbation due to climate change impacts on vectors and reservoirs against a background of socio-economic inequity (Chaves et al., 2008a). Our research has shown that shifts on major ACL mammal reservoir species seem unlikely (Calzada et al., 2015a, González et al., 2015), and that changes in sand fly species composition has not affected dominant vector species (Christensen et al., 1983, Dutari and Loaiza, 2014, Calzada et al., 2013). However, it is still unclear to what degree ACL transmission trends could reflect demographic changes, such as population growth in endemic areas (Alvar et al., 2012) or different degrees in vulnerability to ACL that could be associated with: socio-economic conditions (Chaves et al., 2008a, Chaves et al., 2013, Levins and Lopez, 1999), deforestation (Wijeyaratne et al., 1994), or whether the emergent patterns of ACL transmission echo changes in climate variability (Chaves et al., 2008a, Patz and Olson, 2006).

The large scale of environmental changes and other heterogeneities associated with hypotheses (3) and (4) require coarse-grained macroecological analyses, in the sense that new knowledge can be derived by looking at how the context influences the distribution and abundance of a disease across populations, not individuals (Susser, 1994a, Keyes and Galea, 2014). More specifically, integral variables, i.e., those affecting all or virtually all members of a population (Susser, 1994a), as in the case of climatic phenomena, such as El Niño Southern Oscillation (ENSO) in Panamá, and contextual variables, the mean, median or proportion of an attribute (Susser, 1994a), for example forest cover or poverty within a geopolitical unit, are best studied at the population level. Therefore, this type of study is “macroecological” (Brown, 1995) since the scaling-up of the “ecological” analysis to spatially and temporally relatively coarsely grained scales (Levin, 1992), with all its sacrifice of detail (Brown, 1995), is still helpful to understand patterns of variation (Levins, 1995) in the distribution and incidence of a disease, in the same way the “macroecological” approach has been used to study populations in ecology (Brown, 1995).

Concerning the impacts of climate change on ACL in Central America, in both Costa Rica (Chaves et al., 2008a, Chaves and Pascual, 2006, Chaves and Pascual, 2007, Chaves, 2009) and the Republic of Panamá (Chaves et al., 2014a) interannual cycles in ACL incidence are associated with ENSO. Furthermore, it has also been observed that strong fluctuations in sand fly abundance are associated with ENSO in the Republic of Panamá (Chaves et al., 2014a). Nevertheless, it is unclear how homogeneous/heterogeneous the impacts of ENSO across the Republic of Panamá are, where local variations exist in seasonal weather, the east of the country being slightly more wet than the west (Autoridad Nacional del Ambiente, 2010). Although rainfall is concentrated during the rainy season, which spans from April to December over most of the country, its duration can be slightly shorter towards the west (Autoridad Nacional del Ambiente, 2010). Although temperatures tend to be near constant throughout the country, temperature is slightly lower on the Pacific basin than on the Caribbean basin of the Panamá isthmus (Autoridad Nacional del Ambiente, 2010). Likewise, following global patterns, temperature declines with altitude (Chaves and Koenraadt, 2010). In Panamá, local impacts of ENSO are characterized by a reduction of rainfall over the Pacific coast and an increase of rainfall on the Caribbean coast, during the hot phase (a.k.a., El Niño) of the oscillation, with droughts accentuated during the cold phase (a.k.a., La Niña) of ENSO (Olmedo, 2006). On top of weather heterogeneities, the Republic of Panamá has a wide diversity of natural ecosystems, ranging from dry forests to tropical rainforests, and all the ecosystems have been subjected to different changes in land use over the last 50 years, with heterogeneous deforestation rates rendering a heterogeneous degree of forest cover across the country (Autoridad Nacional del Ambiente, 2010).

The heterogeneity of environmental patterns in Panamá raise questions on whether: (i) ENSO impacts on ACL transmission across the Republic of Panamá are homogenous, and whether patterns are synchronous, with ACL incidence fluctuating in concert across Panamá's Health areas (ii) disease clusters and differential impacts of ENSO are more likely to occur in regions where poverty, or its proxies, are more prominent, (iii) trends in ACL transmission simply reflect population increase and (iv) differential impacts of ENSO, associated or independent of the dominant ecosystem type or contextual forest cover could have led to the clustering of the ACL in certain regions of the country. Here, we study those four questions by analyzing an annual spatio-temporal (1980–2012) dataset of ACL from the 10 health areas in the Republic of Panamá.

## 2 Materials and methods

### 2.1 Data

Annual ACL cases were compiled by health area by the Epidemiology Department of Panamá's Ministry of Health for the period 1980–2012. Briefly data came from a passive surveillance system, where ACL is a disease of compulsory report. Data consist of clinically diagnosed cases (Saldaña et al., 2013), which are often confirmed by microscopic examination of skin lesion scrapings/biopsies, parasite culture, Montenegro skin tests (MST) (Montenegro, 1926) or Indirect Immuno-Fluorescent Agglutination Tests (IFAT) (Miranda et al., 2012). Health areas are the administrative subdivisions of the Panamanian health system, and sometimes comprise more than one province (or indigenous Comarca), or smaller administrative units. Specifically, Panamá province is split into three health areas: Metropolitana (which comprises the metropolitan district of Panamá city and San Miguelito), Oeste (West Panamá province), and Este (East Panamá province and the Comarca Guna de Madugandí). By contrast, the Cocle health area comprises data from the Panamanian provinces of Cocle, Herrera and Los Santos. The Bocas del Toro health area comprises the Bocas del Toro province and Comarca Ngäbe-Bugle, while Darién consists of the Darién province and the Comarcas Emberá-Wounaan and Guna Wargandí. Meanwhile, the following health areas: Veraguas, Chiriquí, Colón and Comarca Guna Yala correspond to the geopolitical provinces/comarcas of the Republic of Panamá with the same name.

Demographic data for the 1990, 2000 and 2010 National censuses of Panamá were obtained from the Dirección de Estadística y Censo of Contraloría General de República de Panamá (http://www.contraloria.gob.pa/inec/). We were able to obtain data on the estimated annual population for each health area, and we also collected data on the % of houses that had: mud walls; thatched roofs; earthen floors and access to piped water. These housing quality data were then used to estimate a health region marginalization-housing quality index, by computing the first principal component, via a principal components analysis (PCA), from a variance covariance matrix (Venables and Ripley, 2002). We chose elements of housing quality because our previous research has indicated this variable as a major driver for the infestation of sand flies and ACL transmission risk, and a good indicator of social marginalization in the Republic of Panamá (Chaves et al., 2013, Saldaña et al., 2013, Calzada et al., 2015a).

For different health areas of Panamá we were also able to estimate dominant climate and life zone indices using maps published by the Autoridad Nacional del Ambiente (ANAM) of Panamá (Autoridad Nacional del Ambiente, 2010). We started by extracting the data from classified raster images (Brunsdon and Comber, 2015) that described the different ecosystems and climates of Panamá using Quantum GIS. With the extracted data expressed as a percent of cover for each health area, we developed a quantitative index following the same methodology used to develop the index for social marginalization-housing quality.

Using data from ANAM we were also able to estimate the % forest cover for each health area using forest cover maps for 1984, 1992 and 2000 (Autoridad Nacional del Ambiente, 2010). For this we digitized the maps and manually classified pixels of area equivalent to 25 km^2^ into natural forest or other use using the software Quantum GIS. Once the raster image was reclassified, we were able to estimate the proportion of land cover by natural forest (which was broadly defined as any type of native vegetation) for all the health areas of Panamá. We were also able to estimate the average precipitation and temperature for each health area employing shapefiles for isoyets and isotherms based on meteorological data from 1970 to 2005, collected by ETESA, Panamá's National Electrical Company.

**Fig. S1:**
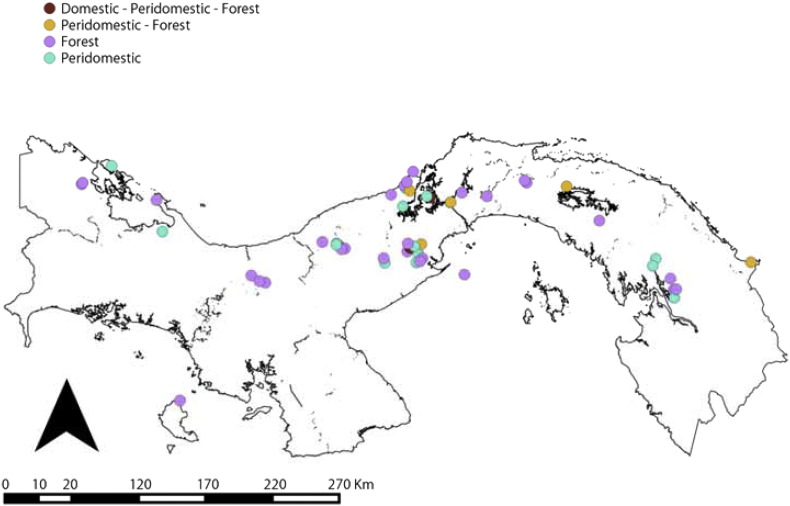
Distribution of habitats sampled for sand fly (Diptera: Psychodidae) presence. See inset legend for classification.

**Fig. S2:**
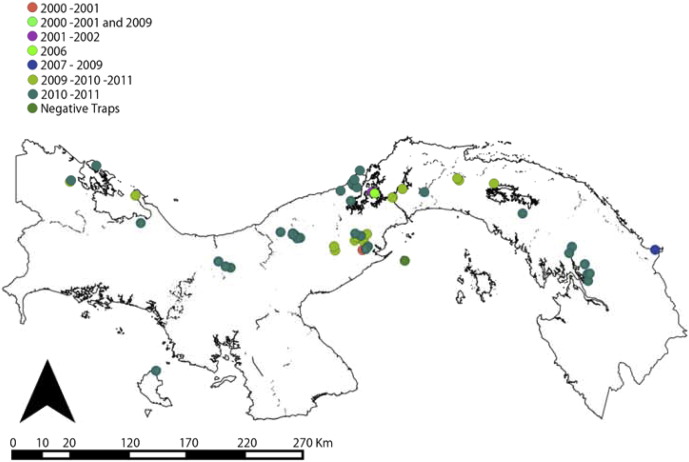
Study period for each sand fly (Diptera: Psychodidae) sampling site. See inset legend for study period. Negative trap indicates places where no *Lutzomyia* sample.

Entomological data came from a series of studies performed by the ICGES and/or Universidad de Panamá. Sand flies were sampled all over the Republic of Panamá with a common standardized method. Briefly, light traps were set at a height of 1.5 to 2.0 m above the ground, overnight (from 6 pm to 6 am), a sampling effort hence referred as trap-night. Habitats were classified following standardized criteria where traps placed inside human dwellings were classified as “domiciliary”. Traps within a 100 m radius from a household were classified as “peridomiciliary”. Traps placed within primary or secondary forests and where no house was in a 100 m radius from the trap were included in the “forest” category. Our sampling effort accounted to a total of 640 trap-night. We collected 12,580 sand flies belonging to *Lutzomyia trapidoi*, *Lu. gomezi* and *Lu. panamensis*. Further details about the entomological sampling have been presented elsewhere (Chaves et al., 2014a). Fig. S1 shows the types of environments where sand flies were caught and Fig. S2 the different years when locations were sampled.

For the temporal analysis of the annual ACL case data, years from 1980 to 2012 were classified in the different phases of El Niño Southern Oscillation (ENSO) following the classification of United States National Oceanic and Atmospheric Administration Climate Prediction Center (http://www.cpc.ncep.noaa.gov/data/indices/ersst3b.nino.mth.81-10.ascii). To homogenize the nomenclature with previous studies, we refer to the years when El Niño occurred as “Niño” and “Niño + 1” to the year following an El Niño event (Bouma and Van der Kaay, 1994, Bouma and Van Der Kaay, 1996, Bouma et al., 1997, Bouma and Dye, 1997). For the sand fly data, which were collected in specific months, we were able to use a more finely grained definition of the ENSO phases, by assigning the extreme high values of the Sea Surface Temperature (SST) 4 anomalies into the “hot” category, the extreme low values into the “cold” category, and everything in between considered as normal (Chaves et al., 2014a, Chaves et al., 2015). To ease the interpretation of the different denominations of the ENSO phases, in general, the “Niño” years were dominated by “hot” months, while the “Niño + 1” are dominated by “cold” months (Bouma and Van der Kaay, 1994, Bouma and Van Der Kaay, 1996, Bouma et al., 1997, Bouma and Dye, 1997), and normal years by data outside the extremes of “hot” and “cold”.

### 2.2 Statistical analysis

#### 2.2.1 Synchrony analysis

We started our analysis by performing a synchrony analysis, where synchrony is expected to occur when disease transmission is not clustered, and can be seen as a null hypothesis, that when rejected, can justify the search for spatio-temporal clusters. To estimate synchrony, which can be formally defined as the degree of concerted fluctuations in annual ACL incidence across the health area time series (Chaves et al., 2012, Ranta et al., 2006, Liebhold et al., 2004), we estimated a correlogram (Ranta et al., 2006, Gouhier and Guichard, 2014). The correlogram is a function that depicts the correlation between time series as function of distance (Ranta et al., 2006). We estimated a Mantel correlogram for the ACL incidence time series (Gouhier and Guichard, 2014). The ACL incidence time series was estimated by dividing the number of reported ACL cases by the total estimated population for health area for each year during the study period. In this procedure, after all the pairwise correlations at time lag zero and across the gradient of distances between the centroids of the health areas were estimated, the statistical significance of the correlogram was tested via a Monte Carlo randomization test where the distance for the correlations was randomly shuffled and the significance of the correlogram from the original data was compared against the ones generated by the Monte Carlo (Bjørnstad and Falck, 2001). For the inference, when the correlogram estimated from the original data was more extreme than the correlograms generated by the Monte Carlo, the null hypothesis that synchrony at a given distance was equal to the average synchrony in the landscape, was rejected (Gouhier and Guichard, 2014).

#### 2.2.2 Cluster analysis

To find spatio temporal clusters of ACL cases we employed the space–time permutation model of Kulldorff et al. (2005). This method is based on the most basic SCAN model where disease counts are studied along a series of circumferences of a given radius. In the space-permutation model the number of cases in a cluster is compared to the expected number of cases assuming independence in the location and times at which cases are counted, and looking at changes in the proportion of cases for a given region. A cluster is defined when the proportion of cases in a geographical area, at a given time, is higher than in other areas (Kulldorff et al., 2005). This method has the advantage of being robust to specific assumptions about the size of the population at risk of acquiring a disease (Kulldorff et al., 2005). For the analysis, we used both circles and ellipses to search for the clusters, and in order to ensure robustness in the results (Levins, 2006). We calculated the geographical centroids of each Panamanian Health area using QGIS.

#### 2.2.3 Spatial analysis of long term averages of ACL incidence rates

We analyzed the association between the average ACL incidence rate from our study period (1980–2012) with the long term averages of temperature and rainfall as well as the climatic and ecosystems index. For this analysis we employed a linear regression (Faraway, 2004) that considered the four covariates mentioned before, and this full model was simplified by a process of backward elimination where variables were removed as long as the Akaike Information Criterion (AIC), a metric for model selection that weights the trade-off between the % of variance explained and the number of covariates, was minimized (Kuhn and Johnson, 2013). We also performed a similar analysis on the average number of ACL cases, as a way to see if transmission heterogeneity could have been influenced by population growth.

#### 2.2.4 ENSO impacts on ACL incidence across regions

The estimated annual ACL incidence rates by health area were used to study the impacts of ENSO on ACL transmission by performing a one way ANOVA (Faraway, 2004). For the ANOVA each year was categorized as Non-El Niño (NON), Niño (a year with an El Niño event) or Niño + 1 (the year following an El Niño event) (Bouma and Dye, 1997). To render each time series stationary, i.e., with a constant mean and variance (Chaves and Pascual, 2006, Shumway and Stoffer, 2011), we included in all models a trend estimated from each ACL incidence time series using the LOWESS method (Venables and Ripley, 2002). We also performed a similar analysis on the ACL cases number, employing negative binomial generalized linear models (NB-GLM) Analysis of Deviance (ANODE), to account for the over-dispersed nature of the ACL case count data (Faraway, 2006), and as a way to see if population growth trends changed the inference about the impacts of ENSO phases on ACL transmission. Assumptions for the ANOVAs and ANODEs were checked, paying special attention to temporal independence, as assessed by temporal Auto-Correlation Functions of the residuals (Shumway and Stoffer, 2011).

#### 2.2.5 Impacts of temporal changes in forest cover and social marginalization on ACL incidence

For this analysis we employed smoothed estimates of ACL annual incidence rate for the years where we were able to estimate forest cover (1984, 1992 and 2000) and the social marginalization index (1990, 2000 and 2010). We smoothed the data with the aim of controlling for artifacts due to sampling in years corresponding to diverse phases of ENSO. For the smoothing we averaged the rates corresponding to the 5 years from 2 years before and 2 years after the years where we had forest cover and social marginalization estimates. The resulting rates were then analyzed using a linear regression, where the ACL rates were log transformed. For forest cover we performed the analysis dividing the health areas in three groups, defined by a preliminary cluster analysis based on *k* neighboring neighbors, knn (Kuhn and Johnson, 2013), since ACL rates and the covariates tended to be clustered.

#### 2.2.6 Regional sand fly abundance patterns

We analyzed the abundance records for each one of the three dominant ACL vector species in Panamá: *Lu. trapidoi*, *Lu. gomezi* and *Lu. panamensis* across the different health areas as function of the following variables: (i) ENSO phase according to the SST (into “cold”, “hot” or “normal”) and (ii) the environment where the sand flies were sampled (For: forest, Per: Peridomicile, Dom: domicile). To account for seasonality in sand fly abundance we run all the models using either the quarter or the month of the observation. For the analysis, we employed, as default, log transformed linear mixed effects models (LMM), and a dummy variable that considered the different sampling locations within a health area as a random factor (Kuhn and Johnson, 2013, Faraway, 2006). However, when there was no health area variability, we employed a linear model (LM). The inference for the LMMs is based on likelihood ratio tests (LRT) and for LMs is based on F-tests. Finally, best models, were chosen based on the minimization of the AIC (Faraway, 2004, Faraway, 2006).

#### 2.2.7 Software

All statistical analyses, with the exception of the SCAN cluster analysis, were performed using the open access software R version 3.2.2. Space–time SCAN permutation clusters were estimated with the software SATSCAN version 9.3. For mapping and raster classification we used the software QGIS version 2.20.

## 3 Results

### 3.1 Synchrony and cluster analysis

**Fig. 1:**
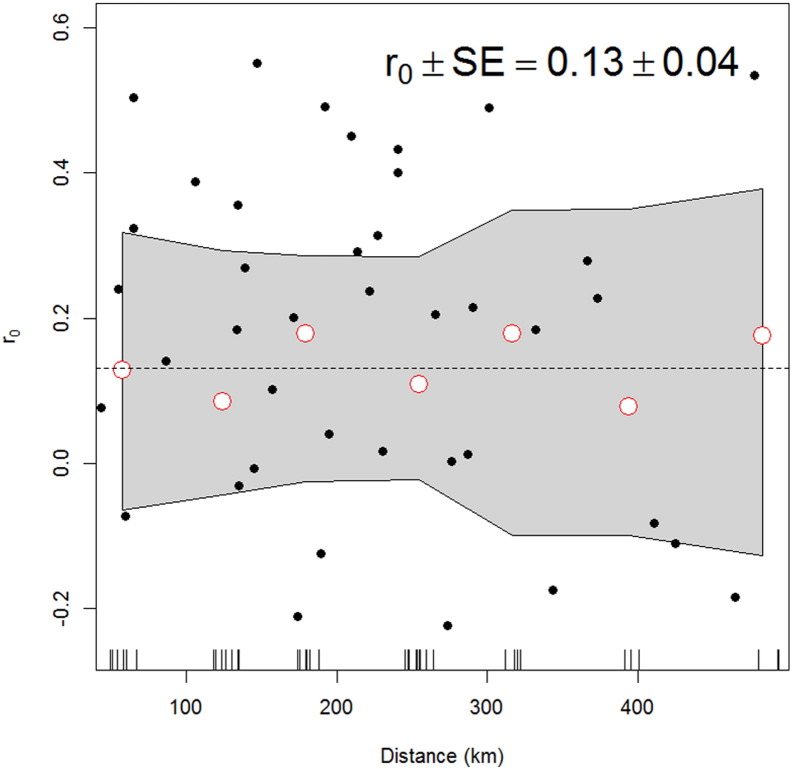
Synchrony of American Cutaneous Leishmaniasis (ACL) incidence as function of the distance between centroids of the health areas of Panamá. Black dots indicate the synchrony between each possible pair of health areas. White dots are average synchrony estimates for a given distance, and the gray area indicates the 95% confidence intervals of the synchrony. For reference the global, or average, synchrony (r_0_) is presented in the figure.

**Fig. 2:**
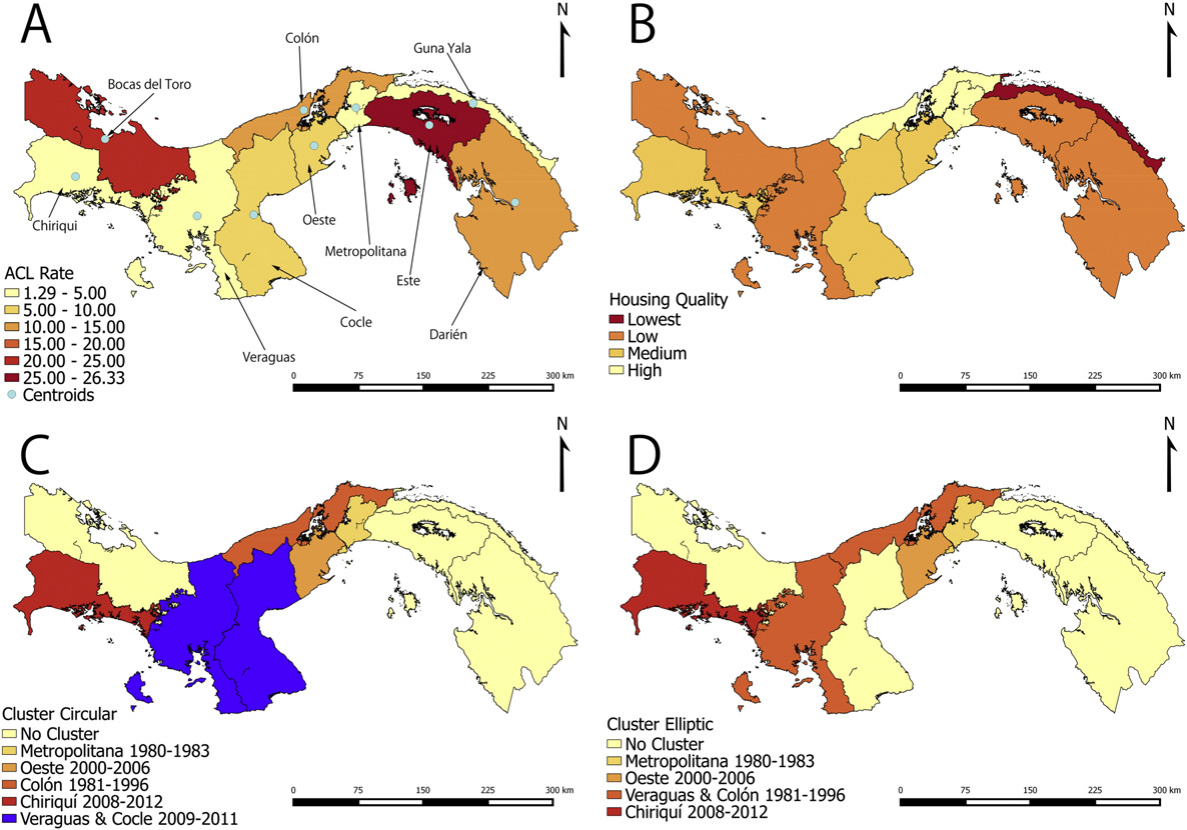
American Cutaneous Leishmaniasis (ACL) incidence rate and clustering in Panamá. (A) Average ACL incidence rate/10,000 people/year (1980–2012) for each health area. Centroids used in subsequent analyses are also presented. (B) Housing quality. (C) Results of the circular SCAN spatio-temporal cluster analysis. (D) Results of the elliptic SCAN spatio-temporal cluster analysis.

Fig. 1 shows the result of the ACL synchrony analysis. It can be observed that although there was no decrease in synchrony as function of distance, the synchrony was not different from 0, indicating that changes in ACL incidence were not concerted across all the health areas of Panamá. Fig. 2A shows the average ACL rate in each health area of Panamá. In Panamá Este, the rate was highest, and it was followed by Bocas del Toro, Colón and Darién. Fig. 2B shows a ranking of the average marginalization-housing quality index for each health area. The marginalization/housing quality index has an interpretation where higher values are associated with poor quality housing and lack of access to piped water (Table S1 in Appendix 1). Fig. 2A and B does not suggest any association between ACL and marginalization/housing quality. Fig. 2C and D shows results of circular and elliptic cluster analysis of ACL cases in each health area. We found five clusters with the circular analysis (Fig. 2C), and four clusters with the elliptic analysis. In the circular analysis we found that Coclé and Veraguas were in the same cluster. But in elliptic analysis, Veraguas was combined with Colón, and Coclé was not included in any cluster. Both the circular (Fig. 2C) and elliptic (Fig. 2D) analyses suggest that ACL transmission clusters moved from east of the Panamá Canal in the early 1980s to the west, especially in the second part of the 2000s.

### 3.2 Spatial analysis of long term averages of ACL incidence rates

**Fig. 3:**
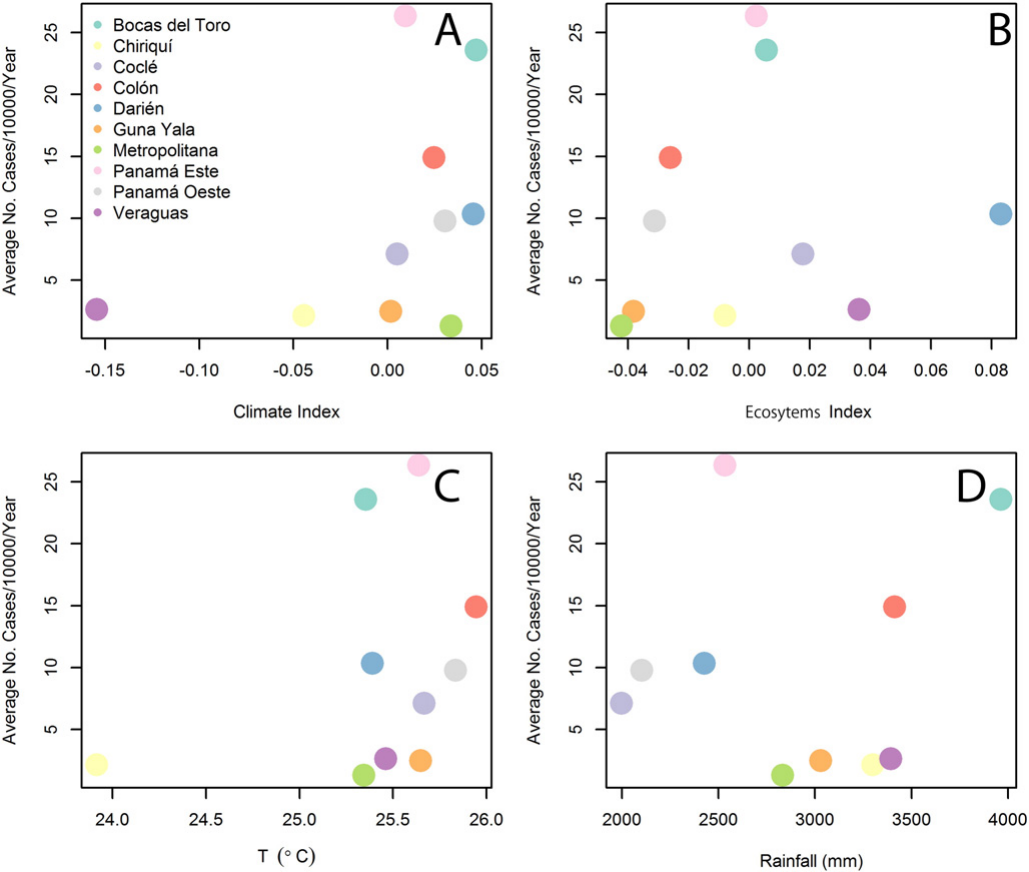
Relationship between the average American Cutaneous Leishmaniasis (ACL) incidence/10,000 people/year (1980–2012) for the health areas of Panamá as function of environmental factors: (A) Climate Index. (B) Ecosystem Index. (C) Temperature. (D) Rainfall.

**Fig. S3:**
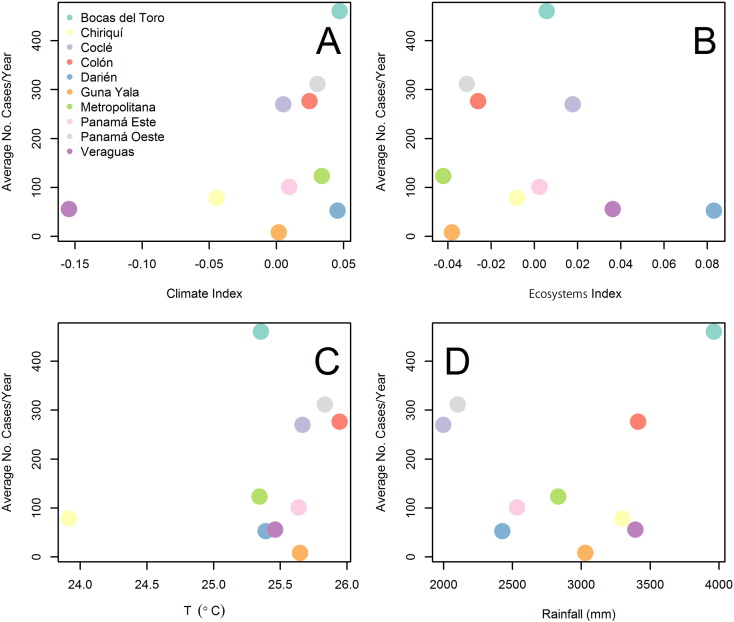
Relationship between the 1980–2012 average American Cutaneous Leishmaniasis (ACL) cases/year and environmental factors. (A) Climate Index. (B) Life Zone Index. (C) Temperature. (D) Rainfall

For the climatic index low values are associated with dry climates and high values associated with wet climates (Table S2 in Appendix 1). For the ecosystems index PCA low values indicate dry ecosystems and high values rainy ecosystems (Table S3 in Appendix 1). Fig. 3 shows the association between the average ACL incidence (1980–2012) and environmental factors. There was a clear positive association between ACL incidence and the climate index (Fig. 3A), temperature (Fig. 3C), and rainfall (Fig. 3D). Although the ecosystem index (Fig. 3B) does not show a clear association with the average ACL incidence, Table 1 shows that parameter estimates for this variable were almost significant when a 2nd degree polynomial was considered. This result suggests that ACL is present in wet/rainy environments of Panamá, a result also observed when examining the average number of ACL cases (Fig. S3). However, the best model (Table S4 in Appendix 1) had an increasing positive association between the number of cases and rainfall (described by the increasing half of u shaped 2nd degree polynomial) and a nearly significant negative association with the ecosystem index, a model that still suggest the importance of wet/rainy environments for ACL transmission.

**Table 1:** Parameter estimates for the best model of the average American Cutaneous Leishmaniasis (1980–2012) Incidence (per 10,000 people/year) as function of environmental factors. This model was selected as best from a full model that also considered rainfall and temperature as covariates (ΔAIC = 1.07).

Factor	Estimate	Std. error	t-Value	Pr (>|t |)
Intercept	1.86	0.25	7.41	0.00031⁎
Climate Type Index	13.86	4.92	2.82	0.03041⁎
Ecosystem Index	1.33	0.81	1.64	0.15
(Ecosystem Index)^2^	− 1.95	0.87	− 2.23	0.07
Multiple R-squared	0.63			

(Ecosystem Index)^2^ indicates the square value of the ecosystem index.

⁎ Statistically significant (P < 0.05).

### 3.3 ENSO impacts on ACL incidence across regions

**Fig. 4:**
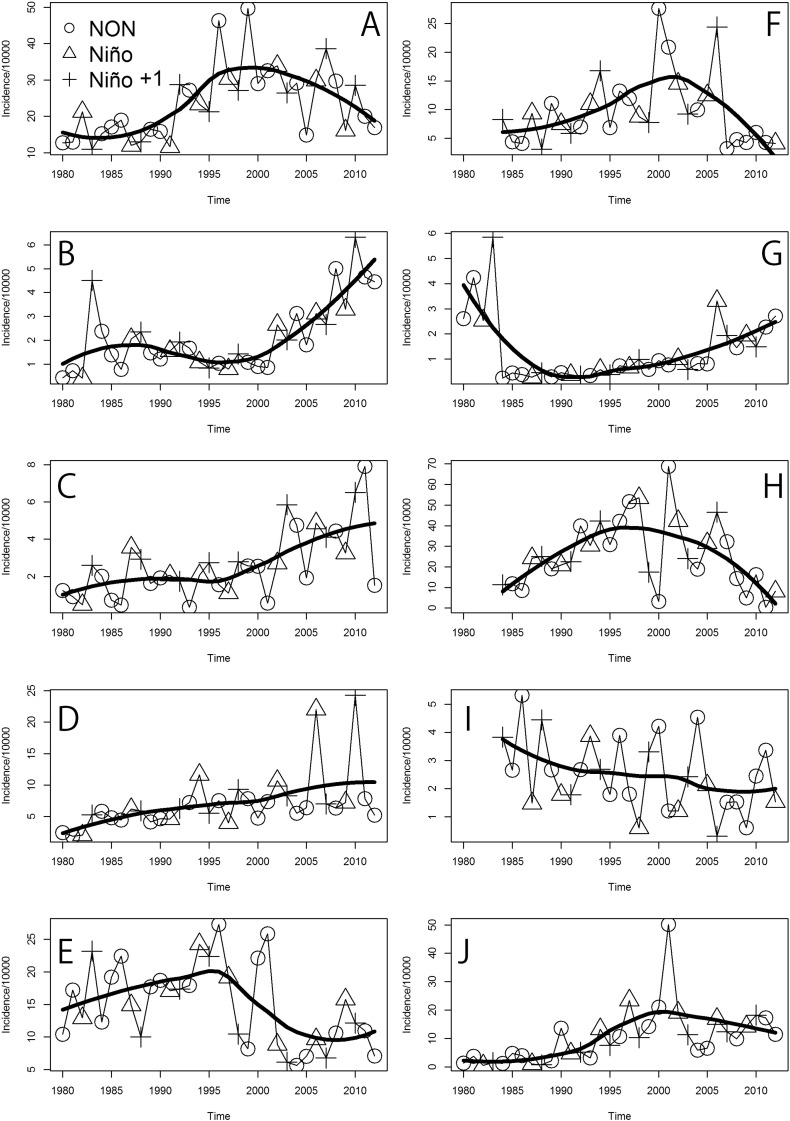
Time series of annual American Cutaneous Leishmaniasis (ACL) incidence for the different health areas of Panamá (1980–2012). Health areas are: (A) Bocas del Toro. (B) Chriqui. (C) Veraguas. (D) Coclé. (E) Colón. (F) Panamá Oeste. (G) Metropolitana. (H) Panamá Este. (I) Guna Yala. (J) Darién. Symbols represent the different El Niño Southern Oscillation (ENSO) phases. In the legend: NON, Niño, and Niño + 1 indicate, respectively, normal years, El Niño event years, and the years following an El Niño event.

**Fig. S4:**
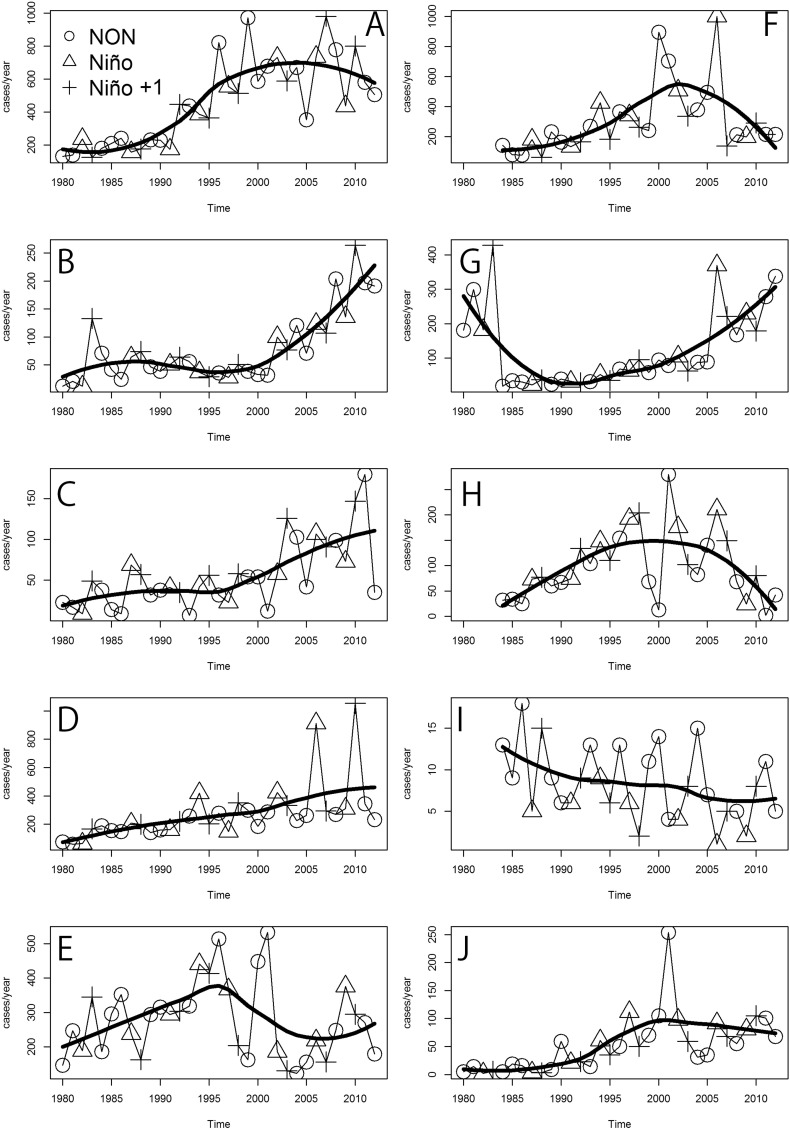
Time series of American Cutaneous Leishmaniasis (ACL) annual cases for the different health areas of Panamá during the different El Niño Southern Oscillation (ENSO) phases. Health areas are: (A) Bocas del Toro. (B) Chriqui. (C) Veraguas. (D) Coclé. (E) Colón. (F) Panamá Oeste. (G) Metropolitana. (H) Panamá Este. (I) Guna Yala (J) Darién. In the legend: NON, Niño, and Niño + 1 indicate, respectively, normal year, El Niño event year, and a year following an El Niño event.

Fig. 4 shows the annual ACL incidence per 10,000 residents from 1980 to 2012, across the different health areas of Panamá. The comparison of Fig. 4 with the raw number of cases (Fig. S4) suggests that increasing trends in ACL are likely independent of population growth and that trends increased in western health areas: Chiriqui (Fig. 4B), Veraguas (Fig. 4C) and Coclé (Fig. 4D). The health areas of Bocas del Toro (Fig. 4A), Panamá Oeste (Fig. 4F), Panamá Este (Fig. 4H) and Darién (Fig. 4J), showed increasing trends of ACL until late 1990s/early 2000s, and then switched into decreasing trends. By contrast, in the Metropolitana health area (Fig. 4G) there was a decreasing trend until the early 1990s, which then changed into an increasing trend. In Guna Yala (Fig. 4I) there was a continuously decreasing trend during the studied period.

Parameter estimates for changes in annual ACL incidence (Fig. 4) as related to ENSO are presented in Table 2. In general, most health areas saw an increase in transmission during the cold ENSO (El Niño + 1) phase (Table 2). However, only in Chiriqui (Fig. 4B) and Veraguas (Fig. 4C), ACL incidence significantly increased (P < 0.05) with the cold ENSO phase (El Niño + 1). By contrast, the cold ENSO phase (Niño + 1) in Panamá Oeste (Fig. 4F) and the hot ENSO phase (Niño) in Guna Yala (Fig. 4L) were associated with a significant decrease (P < 0.05) in transmission when compared with the normal ENSO phase (Non-El Niño years). Also, there were no major differences when a similar analysis was performed on the raw number cases (Table S5 in Appendix 1), the most important difference being that for Coclé the number of cases significantly (P < 0.05) increased during the cold ENSO phase.

**Table 2:** Factors associated with the incidence (per 10,000 people) of American Cutaneous Leishmaniasis in each health area of Panamá (1980–2012). In the table, ENSO indicates the different phases of El Niño Southern Oscillation. Estimated incidence change denotes the incidence difference of normal years (NON) with years when El Niño occurs (Niño) or after it (Niño + 1). Parameter estimates and P-values are based on a one way Analysis of Variance (ANOVA). Panel indicates the panel in Fig. 4.

Panel	Health area	ENSO phase	Estimated incidence change	S.E.	t	P
A	Bocas del Toro	NON	−	−	−	−
		Niño	− 2.88	2.87	− 1.00	0.32
	R^2^ = 0.55	Niño + 1	− 1.05	2.87	− 0.37	0.72
B	Chiriqui	NON	−	−	−	−
		Niño	0.09	0.34	0.25	0.80
	R^2^ = 0.72	Niño + 1	0.84	0.34	2.46	0.0197⁎
C	Veraguas	NON	−	−	−	−
		Niño	0.37	0.53	0.69	0.50
	R^2^ = 0.56	Niño + 1	1.39	0.53	2.62	0.0138⁎
D	Coclé	NON	−	−	−	−
		Niño	2.74	1.64	1.67	0.11
	R^2^ = 0.38	Niño + 1	2.92	1.64	1.78	0.09
E	Colón	NON	−	−	−	−
		Niño	− 12.00	21.70	− 0.55	0.59
	R^2^ = 0.53	Niño + 1	− 15.30	21.70	− 0.71	0.49
F	Panamá Oeste	NON	−	−	−	−
		Niño	1.37	2.08	0.66	0.51
	R^2^ = 0.51	Niño + 1	− 4.59	2.08	− 2.21	0.0359⁎
G	Metropolitana	NON	−	−	−	−
		Niño	0.45	0.37	1.21	0.24
	R^2^ = 0.55	Niño + 1	0.63	0.37	1.71	0.10
H	Panamá Este	NON	−	−	−	−
		Niño	8.41	5.72	1.47	0.15
	R^2^ = 0.52	Niño + 1	6.83	5.72	1.19	0.24
I	Guna Yala	NON	−	−	−	−
		Niño	− 1.52	0.48	− 3.18	0.00382⁎
	R^2^ = 0.42	Niño + 1	− 0.60	0.48	− 1.25	0.22
J	Darién	NON	−	−	−	−
		Niño	0.50	3.10	0.16	0.87
	R^2^ = 0.48	Niño + 1	− 3.17	3.10	− 1.02	0.32

Panel indicates the panel in Fig. 4. NON = Non-El Niño, Niño = a year with an El Niño event, Niño + 1 = the year following an El Niño event.

⁎ Statistically significant (P < 0.05).

### 3.4 Impacts of temporal changes in forest cover and social marginalization on ACL incidence

**Fig. 5:**
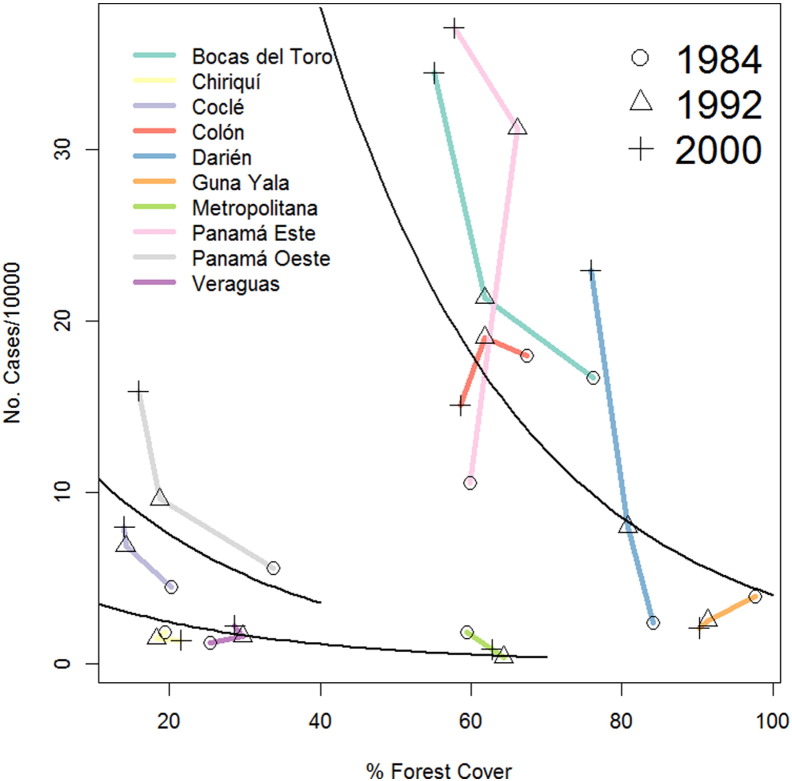
Incidence of American Cutaneous Leishmaniasis (ACL) as function of % forest cover for each health area of Panamá in 1984, 1992, and 2000. Black lines indicate the fitted value for each group. Group A includes Bocas del Toro, Colón, Darién, Guna Yala, Panamá Este. Group B includes Coclé, Panamá Oeste. Group C includes Chiriqui, Veraguas, Metropolitana. Color lines indicate the trajectory of the association for each health area, and symbols indicate the estimate for each year, for details refer to the inset legend. Parameter estimates for the black curves are presented in Table 3.

Fig. 5 shows the association between ACL incidence (cases/10,000/year) and forest cover in each health area. A first glimpse at the raw data suggests a convex relationship, where ACL increases reaching a maximum value at intermediate levels of forest cover and then decreases as forest cover increases past the maximum observed ACL incidence. Nevertheless, at intermediate levels of forest cover (60%), ACL transmission is both maximum and minimum, suggesting that a simple convex relationship is not the most appropriate to describe the association between forest cover and ACL incidence. Moreover, for health areas with relatively low forest cover, a common pattern is that ACL incidence decreases with increasing forest cover. Thus, based on a knn cluster analysis, we divided health areas into three groups: Group A (Bocas del Toro, Colón, Darién, Guna Yala, Panamá Este are included), where health areas have a high proportion of forest cover and the highest ACL incidence rates; Group B (Coclé and Panamá Oeste are included), where health areas have a medium to low forest cover and Group C (Chiriqui, Veraguas and Metropolitana are included), which had the lowest forest cover. This analysis suggests that ACL incidence decreased with forest cover, although the magnitude of the change is conditioned on the proportion of forest cover and the incidence (Table 3). Parameter estimates from a model considering the three groups are shown in Table 3, which show that in all groups ACL incidence decreases with forest cover at the same rate, yet baseline transmission increased with forest cover. Table 3 also shows that variance in the health areas was almost as big as the error, indicating an important degree of heterogeneity in ACL incidence was present among the different health areas.

**Table 3:** Parameter estimates for a model of American Cutaneous Leishmaniasis incidence as function of Forest Cover. Group A includes Bocas del Toro, Colón, Darién, Guna Yala, Panamá Este, Group B includes Coclé and Panamá Oeste, Group C Chiriqui, Veraguas and Metropolitana. The data and model fit are presented in Fig. 5.

Factor	Estimate (Difference from Group A)	Std. error	t-Value	95% CL
Group A	5.14	0.74	6.97⁎	3.844, 6.404
Group B	2.77 (− 2.37)	0.65	− 3.64⁎	− 3.481, − 1.233
Group C	1.65 (− 3.49)	0.49	− 7.10⁎	− 4.333, − 2.639
Forest cover slope	− 0.04	0.01	− 3.84⁎	− 0.054, − 0.020
Variance health area	0.14			
Variance error	0.25			

⁎ Statistically significant (P < 0.05).

**Fig. 6:**
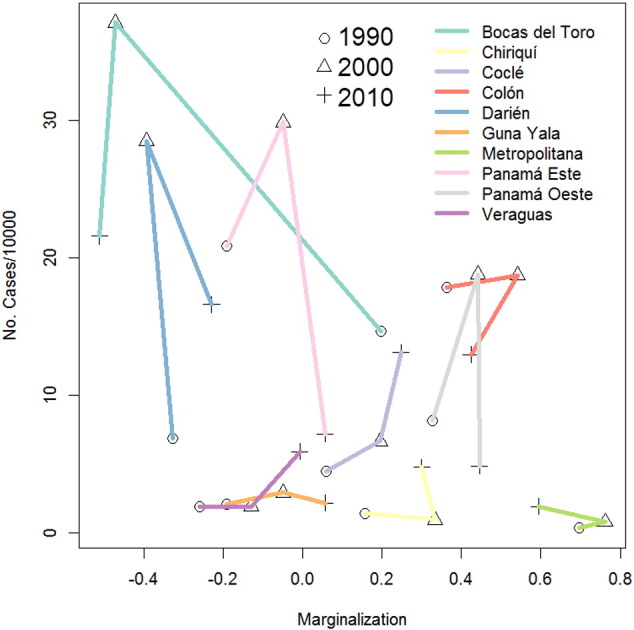
Incidence of American Cutaneous Leishmaniasis (ACL) as function of socio economic marginalization for each health area of Panamá in 1990, 2000, and 2010. Color lines indicate the trajectory of the association for each health area, and symbols represent the estimate for each year, for details refer to the inset legend.

Fig. 6 shows the associations between average ACL incidence (cases/10,000/year) in each health area and socio-economic marginalization as measured by housing quality, the figure suggests no clear association pattern, a result furtherly confirmed by a regression analysis (Table S6 in Appendix 1).

### 3.5 Regional sand fly abundance patterns

**Fig. 7:**
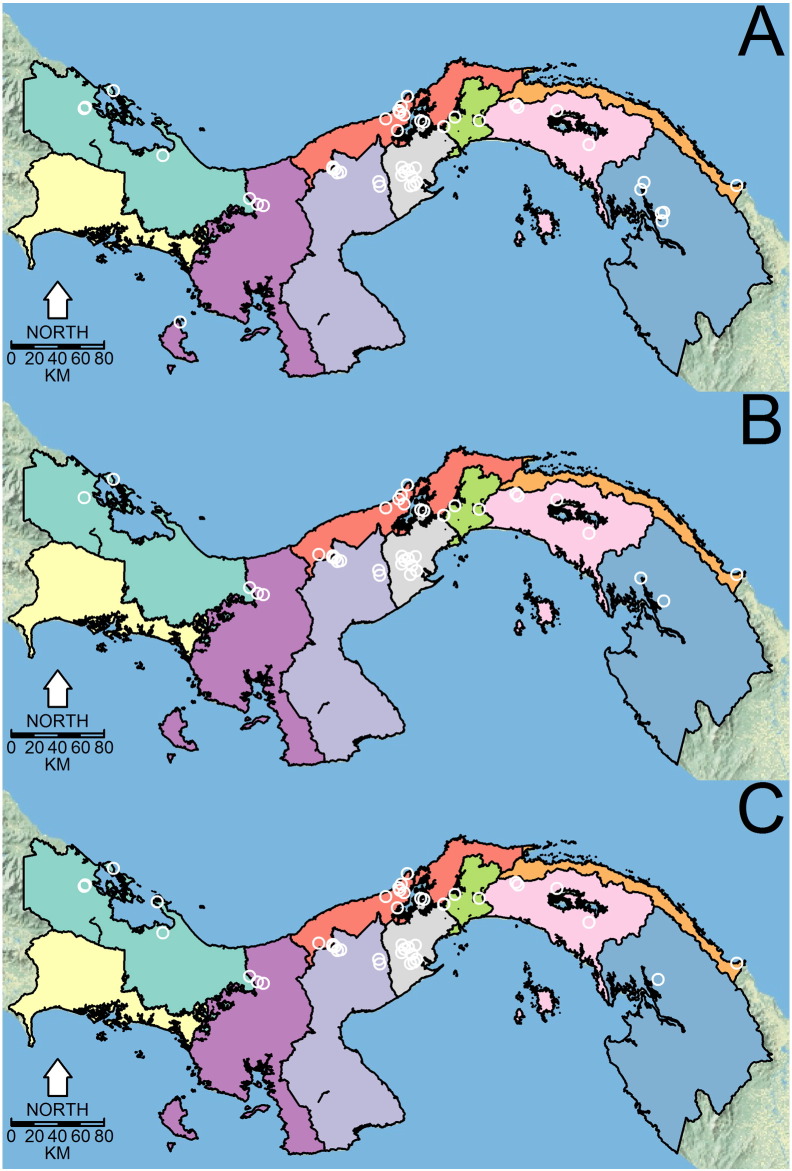
Distribution of dominant *Lutzomyia* spp. vectors of American Cutaneous Leishmaniasis in Panamá. (A) *Lutzomyia gomezi*. (B) *Lutzomyia trapidoi*. (C) *Lutzomyia panamensis*. White circles indicate the study sites where each species was collected. Further details about the sampling time and environment are presented, respectively, in Figs. S3 and S4. Distribution of dominant *Lutzomyia* spp. vectors of American Cutaneous Leishmaniasis in Panamá. (A) *Lutzomyia gomezi*. (B) *Lutzomyia trapidoi*. (C) *Lutzomyia panamensis*. White circles indicate the study sites where each species was collected. Further details about the sampling time and environment are presented, respectively, in Figs. S1 and S2.

Fig. 7 shows the distribution of dominant *Lutzomyia* spp. vectors of ACL in Panamá. *Lu. gomezi* and *Lu*. *trapidoi* have been more frequently caught in Eastern Panamá. Across health areas, the sampling of forest habitats was more common than domiciliary or peridomiciliary habitats (Fig. S1). Similarly, there is a gap of knowledge about sand flies in Chiriqui and Guna Yala (Table 4, Fig. 7). Table 4 also shows an abridged version of all the analyses we performed, showing that in general, *Lu. gomezi*, *Lu. trapidoi* and *Lu. panamensis* decreased their abundance during the cold phase of ENSO. A more detailed account of the significance of the parameters backing the results of Table 4 are presented as supplementary materials for *Lu. trapidoi* (Table S7 in Appendix 1), *Lu. gomezi* (Table S8 in Appendix 1) and *Lu. panamensis* (Table S9 in Appendix 1), and the parameters for the models for each species and health area with enough data for an analysis are presented in Appendix 1 (Tables S10, S11, S12, S13, S14, S15, S16, S17, S18, S19, S20, S21, S22, S23, S24 and S25).

**Table 4:** Association between the number of collected sand flies and Sea Surface Temperature (SST) changes in each health area.

Vector Species	*Lutzomyia trapidoi*		*Lutzomyia gomezi*		*Lutzomyia panamensis*	
Health Area/SST	Hot	Cold	Hot	Cold	Hot	Cold
Bocas Del Toro	N.S.	N.S.	N.S.	N.S.	Seasonal	Seasonal
Chiriquí	N.D.	N.D.	N.D.	N.D.	N.D.	N.D.
Coclé	Same as normal	Reduction	Same as normal	Reduction	Increase	Reduction
Colón	Same as normal	Reduction	Same as normal	Reduction	Same as normal	Reduction
Darién	N.S.	N.S.	Same as normal	Increase	N.S.	N.S.
Guna Yala	N.E.S.	N.E.S.	N.E.S.	N.E.S.	N.E.S.	N.E.S.
Metropolitana	Same as normal	Same as normal	Same as normal	Same as normal	Same as normal	Same as normal
Panamá Este	Increase	Reduction	Increase	Reduction	Increase	Reduction
Panamá Oeste	N.S.	N.S.	Same as normal	Reduction	Same as normal	Reduction
Veraguas	N.S.	N.S.	Seasonal	Seasonal	Seasonal	Seasonal

N.S. = not sampled, N.D. = no data, N.E.S. = not enough samples, Seasonal = only seasonality was significant.

## 4 Discussion

The macroecological approach has the potential to offer new insights about the processes shaping disease transmission patterns. Nevertheless, some limitations are common to this large-scale approach, starting with the quality of data at hand for large geographical scales, which, although systematically collected, are likely biased by coming from clinical manifestations observed at health centers, as it is the case for the data this study used. Moreover, some emergent patterns at broad geographical scales do not necessarily reflect processes occurring at the “local” scale at which transmission events occur (Susser, 1994a, Susser, 1994b). It should also be noted that inferences do not extrapolate to “individual level” risk factors of transmission as that would be an “ecological fallacy” (Susser, 1994a, Susser, 1994b). Nevertheless, as recognized in ecology, the vantage point of “macroecology” (Brown, 1995) is a necessary complement to better understand the drivers of spatio-temporal heterogeneity in disease transmission. In that sense our results from Panamá suggest that impacts of ENSO on ACL transmission seem to be homogenous across the southern part of Central America, where interannual increases in transmission are associated with the cold phase of ENSO (Chaves et al., 2008a, Chaves et al., 2014a, Chaves and Pascual, 2006). Moreover, although transmission was not synchronous across the whole Republic of Panamá because of the moving nature of clusters around the Panamá canal, there was a common pattern of increased transmission across the health areas of Panamá during the cold phase of ENSO, a pattern similar to what we found when looking at monthly incidence records for the whole country during 2000–2010 (Chaves et al., 2014a).

For Panamá, it has also been observed that malaria transmission peaks during the cold ENSO phase (Hurtado et al., 2014). In neighboring Costa Rica similar increases have been observed for snakebites (Chaves et al., 2015) and dengue fever (Fuller et al., 2009). Interestingly, similar mechanisms may be playing a role in the cycles of all these diseases, since for other vector-borne diseases (malaria and dengue fever) weather changes associated with ENSO might trigger outbreaks of vectors (Chaves et al., 2014b, Chaniotis et al., 1971), which might be associated with subsequent increases in transmission (Chaves et al., 2014a, Smith et al., 2010). The relationship of vector abundance with transmission deserves further attention, since one counter-intuitive pattern is that transmission increases when vector abundance decreases across the health areas of Panamá. One possibility is that disease outbreaks could be associated with outbreaks in vector populations (Chaves et al., 2014a). But in addition, the counter-intuitive changes might also be related to the relative long delay in the clinical manifestation of ACL (Chaves, 2009). Nevertheless, further field studies at the local scale of transmission could help to better understand the nature of this pattern. The zoonotic nature of ACL could also play a role in ACL cycles, since cycles on reservoir abundance associated with changes in plant productivity (Adler, 1998, Wright et al., 1999) might increase the abundance of competent reservoirs and transmission (Lima et al., 1999, Kausrud et al., 2008). Indeed, changes in the abundance of mammals that serve as reservoirs for ACL has been suggested as a driver of interannual cycles on snakebites, which also have been reported to increase during the cold phase of ENSO (Chaves et al., 2015).

The association between ACL and humid environments has been well documented in the Republic of Panamá (Christensen et al., 1983). This is related to the environmental requirements of sand flies, which, in general, thrive in humid environments (Rutledge and Ellenwood, 1975a, Rutledge and Ellenwood, 1975b, Rutledge and Ellenwood, 1975c, Rutledge and Mosser, 1972). This association could also explain low ACL transmission in the dry health area of Coclé. The recent clustering of ACL transmission in the border with Costa Rica is important to understand the regional eco-epidemiology of the disease. On the Costa Rican side of the border, the disease is also clustered (Chaves et al., 2008a). Knowledge about vectors in this border is relatively scarce, for example, as our analysis shows, no sand flies have been collected in Chiriquí over recent years, and information about vectors on the Costa Rican side of the border is also scarce (Chaves et al., 2008a). In that sense, a research priority is the characterization of ACL eco-epidemiology in Bocas del Toro and Chiriquí, and if possible in a joint effort with Costa Rica, given the high mobility of populations across the border, specially of Ngöbe natives (Hurtado et al., 2014, Calzada et al., 2015b, Cáceres et al., 2012), in the area.

The lack of association between the housing quality index and ACL transmission might reflect many situations, first it can be an example of the opposite to an “ecological fallacy”, where patterns observed across individual units of analysis at the local scale of transmission are not significant at the population level across regions (Susser, 1994a, Susser, 1994b). Specifically, the local pattern of transmission, where housing quality is a significant transmission risk when comparing households (Chaves et al., 2013), was not informative about transmission patterns at the health area scale. Second, it might be related to data quality. For example, ACL incidence rates have been steadily decreasing in the Guna Yala autonomous Comarca, but this might reflect the fact that the population has grown at a rate that outpaces the growth of health services (Hurtado et al., 2014). This situation contrasting to what has been observed in neighboring Costa Rica, where ACL is more common across poor communities (Chaves et al., 2008a). Nevertheless, independently of the possibility of low quality data, other factors might be at play. For example, a detailed study of leishmaniasis in Guna Yala, mentioned that clinical ACL cases in native Guna populations of this comarca were uncommon (Christensen et al., 1972). Similarly, epidemiological surveys in Eastern Panama showed that clinical ACL cases were rare (low incidence) in indigenous people when compared with other populations of Panamá (Christensen et al., 1999). Thus, if the health system coverage was similar to that of other health areas in Panamá, it would not be surprising that clinical ACL cases were still low in Guna Yala.

In contrast, a more clear pattern was observed when studying ACL incidence as function of forest cover, where in general, incidence decreased with forest cover, a pattern also observed in Costa Rica (Chaves et al., 2008a). This pattern can be related to a “dilution effect” (Civitello et al., 2015), where a larger forest cover implies a higher biodiversity and a decrease in the risk of transmission (Chaves et al., 2007, Chaves et al., 2008b). This pattern might also be related to changes in the community of sand fly vectors, whose species diversity decrease in transformed environments, in a way where vectors with higher vectorial capacity become dominant (Chaves, 2011, Chaves and Añez, 2004, Chaves and Añez, 2016). To better understand the mechanism giving raise to this pattern, it is necessary to perform studies where infection on vertebrate hosts and sand flies are studied simultaneously across a forest cover gradient, at the local scale of transmission. From a theoretical perspective, the pattern where ACL incidence decreases as function of forest cover in areas with similar forest cover, yet it has an overall convex relationship with forest cover, reaching a maximum at intermediate levels of forest cover, requires the proposal of mathematical models that can consider catastrophic shifts (Scheffer, 2009) in the relationship between forest cover and ACL transmission. Non-linear mathematical models can easily explain this type of patterns (Scheffer et al., 2001), and help to better grasp the relationship between forest cover and the transmission of ACL in a way that could help to propose sound ways of ecosystem modification that minimize the risk of emergence for ACL and other similar vector-borne diseases (Levins et al., 1994).

Finally, our study showed how a “macroecological” approach can be useful to identify knowledge gaps in the eco-epidemiology of a vector-borne disease, and how in general the impacts of ENSO on sand flies and ACL were mainly similar across the Republic of Panamá, yet transmission hotspots changed through our study period, and our results indicate, that as observed in neighboring Costa Rica (Chaves et al., 2008a), high forest cover is associated with a decrease in ACL at coarsely grained geographical scales.

The following are the supplementary data related to this article.

Supplementary material. Image 1
