# Supplementary material for: Macroecological patterns of American Cutaneous Leishmaniasis transmission across the health areas of Panamá (1980–2012)
Source: Parasite Epidemiol Control. 2016 Mar 18;1(2):42–55. doi: 10.1016/j.parepi.2016.03.003 (PMC5991822; doi:10.1016/j.parepi.2016.03.003)
Supplement: Supplementary file 1 — Supplementary material. [file mmc1.pdf]

Appendix 1

This appendix contains tables for all the supplementary analyses associated with the article. Briefly, Tables S1, S2 and S3 report results for the principal component analyses used to build different indices that reduced the dimensions of several associated variables. Tables S4 and S5 report results of analyses performed on American Cutaneous Leishmaniasis (ACL) case counts as opposed to the ACL rates reported in the main manuscript. Table S6 reports the non-significant results of the analysis about the relationship between marginalization and American Cutaneous Leishmaniasis rates. Tables S7, S8 and S9 report the factors significantly associated with the abundance of the different dominant sand fly vector species in Panamá. Finally, Tables S10 to S25 report parameter estimates used to build Table 4 in the main article.

Table S1 Principal Component Analysis for the estimation of a marginalization index. Columns indicate the loadings for each component.

Year 1990	Comp.1	Comp.2	Comp.3	Comp.4
Mud Walls	0.53	-0.44	0.37	0.62
Thatched Roofs	0.55	0.71	0.39	-0.20
Earthen Floors	0.53	-0.48	-0.21	-0.66
Access to Piped Water	-0.36	-0.26	0.82	-0.36
% Variance	0.86	0.07	0.05	0.02
Cumulative Variance	0.86	0.93	0.98	1.00
**Year 2000**	**Comp.1**	**Comp.2**	**Comp.3**	**Comp.4**
Mud Walls	0.45	-0.57	-0.37	0.58
Thatched Roofs	0.51	0.00	0.84	0.18
Earthen Floors	0.46	-0.38	0.00	-0.80
Access to Piped Water	-0.57	-0.73	0.39	0.00
% Variance	0.89	0.07	0.04	0.01
Cumulative Variance	0.89	0.96	0.99	1.00
**Year 2010**	**Comp.1**	**Comp.2**	**Comp.3**	**Comp.4**
Mud Walls	0.53	-0.52	-0.29	0.61
Thatched Roofs	0.53	0.00	0.83	-0.13
Earthen Floors	0.50	-0.11	-0.44	-0.74
Access to Piped Water	-0.43	-0.85	0.16	-0.27
% Variance	0.91	0.07	0.01	0.00
Cumulative Variance	0.91	0.98	1.00	1.00

Table S2 Principal Component Analysis for the estimation of a climate type index. Columns indicate the loadings for each component.

Climate Type	Comp.1	Comp.2	Comp.3	Comp.4	Comp.5	Comp.6	Comp.7
Low Mountain Tropical	0.00	-0.11	0.22	-0.47	0.83	0.00	0.16
Tropical with a long dry season	0.00	0.96	-0.17	0.00	0.18	0.00	0.00
Sub Equatorial with a dry season	-0.98	0.00	0.00	0.00	0.00	0.14	0.11
Low Mountain Oceanic	0.00	0.00	0.26	0.75	0.33	-0.50	0.00
Tropical Oceanic	-0.17	-0.14	-0.46	-0.26	0.00	-0.66	-0.48
Middle and High Mountain Tropical	0.00	0.00	0.11	0.25	0.27	0.45	-0.81
Tropical Oceanic with a short dry Season	0.00	-0.18	-0.79	0.30	0.30	0.30	0.26
% Variance	0.66	0.23	0.07	0.02	0.01	0.00	0.00
Cumulative Variance	0.66	0.89	0.96	0.99	1.00	1.00	1.00

Table S3 Principal Component Analysis for the estimation of a Life Zone index. Columns indicate the loadings for each component.

Life Zone	Comp.1	Comp.2	Comp.3	Comp.4	Comp.5	Comp.6
Humid	0.84	0.46	-0.20	0.19	0.00	0.00
Very Humid	0.51	-0.61	0.17	0.59	0.00	0.00
Low Mountain Rainy	0.00	-0.20	0.10	-0.23	0.93	0.16
Premountain Rainy	0.18	-0.52	0.19	-0.75	-0.32	0.00
Mountain Rainy	0.00	0.00	0.00	0.00	0.15	-0.99
Dry	0.00	0.33	0.94	0.00	0.00	0.00
% Variance	0.17	0.17	0.17	0.17	0.17	0.17
Cumulative Variance	0.17	0.33	0.50	0.67	0.83	1.00

Table S4 Parameter estimates for the best model of the average number of American Cutaneous Leishmaniasis (1980-2012) cases as function of environmental factors. This model was selected as best from a full model that also considered the climate type index and temperature as covariates (ΔAIC=-0.32)

Factor	Estimate	Std.Error	t-value	Pr(>|t|)
Intercept	3050.79	483.34	6.31	0.000738*
Rainfall	-2089.30	339.52	-6.15	0.000844*
$(Rainfall)^2$	362.70	57.77	6.28	0.0007.59*
Life Zone Index	-116.89	55.82	-2.09	0.08
Multiple R-squared	0.87			

*Statistically significant (P<0.05), $(Rainfall)^2$ indicates the square value of the rainfall.

Table S5 Factors associated with the number of American Cutaneous Leishmaniasis cases in each health area of Panamá. For the analysis, we employed, a negative binomial generalized linear models (NB-GLM). Parameter estimates and p-values are based on an Analysis of Deviance (ANODE). In the table, ENSO indicates the different phases of El Nino Southern Oscillation, and overdispersion denotes the NB dispersion parameter. Panel indicates the corresponding panel for the raw case data in Figure S4,

Panel	Health Area	ENSO	Rate Change	Parameter Estimate	S.E.	Z	P
A	Bocas del Toro	NON	1.00	-	-	-	-
		Niño	0.90	-0.11	0.11	-0.98	0.33
		Niño+1	0.97	-0.03	0.11	-0.27	0.78
		Overdispersion	-	15.56	3.96	-	-
B	Chiriqui	NON	1.00	-	-	-	-
		Niño	1.07	0.07	0.16	0.44	0.66
		Niño+1	1.53	0.43	0.16	2.70	0.0060*
		Overdispersion	-	8.43	2.40	-	-
C	Veraguas	NON	1.00	-	-	-	-
		Niño	1.21	0.19	0.21	0.93	0.35
		Niño+1	1.71	0.54	0.21	2.60	0.0090*
		Overdispersion	-	4.76	1.31	-	-
D	Coclé	NON	1.00	-	-	-	-
		Niño	1.34	0.29	0.15	1.90	0.06
		Niño+1	1.40	0.33	0.15	2.20	0.0289*
		Overdispersion	-	8.15	2.03	-	-
E	Colón	NON	1.00	-	-	-	-
		Niño		0.00	0.14	-0.03	0.98
		Niño+1		-0.14	0.14	-0.99	0.32
		Overdispersion	-	9.62	2.42	-	-
F	Panamá Oeste	NON	1.00	-	-	-	-
		Niño	1.11	0.11	0.17	0.62	0.54
		Niño+1	0.60	-0.51	0.18	-2.90	0.004*
		Overdispersion	-	7.11	1.88	-	-
G	Metropolitana	NON	1.00	-	-	-	-
		Niño	1.38	0.32	0.21	1.60	0.12

		Niño+1	1.39	0.33	0.21	1.60	0.11
		Overdispersion	-	4.64	1.18	-	-
H	Panamá Este	NON	1.00	-	-	-	-
		Niño	1.18	0.17	0.28	0.60	0.55
		Niño+1	1.21	0.19	0.28	0.69	0.49
		Overdispersion	-	2.83		-	-
I	Guna Yala	NON	1.00	-	-	-	-
		Niño	0.49	-0.72	0.19	-3.70	>0.001*
		Niño+1	0.80	-0.22	0.16	-1.40	0.17
		Overdispersion	-	-	-	-	-
J	Darién	NON	1.00	-	-	-	-
		Niño	0.83	-0.19	0.24	-0.77	0.44
		Niño+1	0.64	-0.45	0.24	-1.80	0.07
		Overdispersion	-	3.63	1.00	-	-

Panel indicates the panel in Figure 4. NON = Non El Niño, Niño = a year with an El Niño event, Niño +1 = the year following an El Niño event. *Statistically significant (P<0.05)

Table S6 Parameter estimates for a model of American Cutaneous Leishmaniasis incidence as function of socio-economic marginalization.

Factor	Estimate	Std.Error	t-value	95% CL
Intercept	1.79	0.36	4.98	1.050, 2.527*
Variance Health Area	1.16			
Variance Error	0.39			

*Statistically significant (P<0.05)

Table S7 Factors associated with *Lutzomya trapidoi* abundance in the different health areas of Panamá. For the analysis, we employed, as default, log transformed linear mixed effects models (LMM). However, when there was no health area variability, we employed a linear model (LM). In the table for LMMs we present likelihood ratio test estimates (LRT) and the p-values are based on a Chi square test. For LMs we report the F-Values and P-values are based on a F-test. For explanation about factors and labels, please refer to the main text.

Health Area	Factor	Levels			
			DF	F value	Pr(>F)
Bocas del Toro	Environment	Per,For	1	4.73	<0.05
	Quarter	All	3	1.30	0.32
			DF	F value	Pr(>F)
Veraguas	Quarter	First,Second,Fourth	2	1.67	0.24
			DF	LRT	Pr(Chi)
Coclé	Environment	Per,For	1	2.92	0.09
	Month	Jan,Mar,Jul,Aug	3	17.92	<0.0004*
	SST	All	2	13.13	<0.001*
			DF	LRT	Pr(Chi)
Colón	Environment	Per,For	1	3.82	<0.05
	Month	Jan,Feb,Mar,Apr,May,Aug,,Sep,Oct,Nov	8	19.17	<0.01*
	SST	All	2	13.91	<0.0009*
			DF	LRT	Pr(Chi)
Panamá Oeste	Environment	All	2	1.68	0.43
	Month	All	11	51.73	<0.000

					1*
	SST	All	2	1.56	0.46
			DF	F value	Pr(>F)
Metropolitana	Quarter	All	3	1.65	0.26
	SST	Hot,Cold	1	1.94	0.21
			DF	LRT	Pr(Chi)
Panamá Este	Environment	Per,For	1	0.47	0.49
	Month	Jan,Feb,Mar,Aug,Sep	4	7.28	0.12
	SST	All	2	7.88	<0.01*
			DF	F value	Pr(>F)
Darién	Environment	Per,For	1	0.03	0.87
	SST	All	2	0.99	0.39

SST= Sea Surface Temperature, Per= Peridomicile, For= Forest

*Statistically significant (P<0.05)

Table S8 Factors associated with *Lutzomyia gomezi* abundance in the different Health Areas of Panamá. For the analysis, we employed, as default, log transformed linear mixed effects models (LMM). However, when there was no health area variability, we employed a linear model (LM). In the table for LMMs we present likelihood ratio test estimates (LRT) and the p-values are based on a Chi square test. For LMs we report the F-Values and P-values are based on a F-test. For explanation about factors and labels, please refer to the main text.

Health Area	Factor	Levels			
			DF	LRT	Pr(Chi)
Bocas del Toro	Environment	Per,For	1	1.56	0.21
	Quarter	All	3	6.73	0.08
	SST	All	2	2.56	0.28
			DF	F-value	Pr(>F)
Veraguas	Season	All	1	5.09	<0.04*
	SST	Hot,Cold	1	2.83	0.12
			DF	LRT	Pr(Chi)
Coclé	Environment	Per,For	1	0.05	0.83
	Month	Jan,Mar,Jul,Aug	3	24.00	<0.0001*
	SST	ALL	2	16.56	<0.0002*
			DF	LRT	Pr(Chi)
Colón	Environment	For,Per	1	0.05	0.83
	Month	Jan,Feb,Mar,Apr,May,Aug,Sep,Oct,Nov	8	24.00	<0.0001*
	SST	All	2	16.56	<0.0002*
			DF	LRT	Pr(Chi)
Panamá Oeste	Environment	All	2	5.34	0.07
	Quarter	All	3	11.46	<0.009*
	SST	All	2	9.69	<0.007

*

			DF	F value	Pr(>F)
Metropolitana	Environment	Per,For	1	0.19	0.68
	Month	Feb,Mar,Apr,Jul, Aug,Sep,Oct,Nov	7	1.89	0.28
	SST	Hot,Cold	1	0.45	0.54
			DF	F value	Pr(>F)
Panamá Este	Environment	For,Per	1	0.46	0.51
	Month	Jan,Feb,Mar,Aug, Sep	4	1.97	0.14
	SST	All	2	5.90	<0.01*
			DF	LRT	Pr(Chi)
Darién	Environment	For,Per	1	1.07	0.30
	Season	All	1	26.45	<0.0001*
	SST	All	2	41.72	<0.0001*

SST= Sea Surface Temperature, Per= Peridomicile, For= Forest

*Statistically significant (P<0.05)

Table S9 Factors associated with *Lutzomyia panamensis* abundance in the different Health Areas of Panamá. For the analysis, we employed, as default, log transformed linear mixed effects models (LMM). However, when there was no health area variability, we employed a linear model (LM). In the table for LMMs we present likelihood ratio test estimates (LRT) and the p-values are based on a Chi square test. For LMs we report the F-Values and P-values are based on a F-test. For explanation about factors and labels, please refer to the main text.

Health Area	Factor	Levels			
			DF	LRT	Pr(Chi)
Bocas del Toro	Environment	Per,For	1	1.18	0.28
	Quarter	All	3	8.36	<0.03*
	SST	All	2	5.61	0.06
			DF	LRT	Pr(Chi)
Veraguas	Quarter	First,Second,Fourth	2	6.65	<0.03*
			DF	LRT	Pr(Chi)
Coclé	Environment	For,Per	1	0.11	0.74
	Month	Jan,Mar,Jul,Aug	3	18.24	<0.0003*
	SST	Hot,Cold	1	4.45	<0.03*
			DF	LRT	Pr(Chi)
Colón	Environment	For,Per	1	8.04	0.004569*
	Quarter	All	3	33.69	<0.0001*
	SST	All	2	48.29	<0.0001*
			DF	LRT	Pr(Chi)
Panamá Oeste	Environment	All	2	4.58	0.10
	Month	All	11	53.18	<0.0001*
	SST	All	2	11.08	<0.003*
			DF	F value	Pr(>F)
Metropolitana	Month	Feb,Mar,Apr,Jul,Aug,Oct,Nov	6	1.86	0.39

	SST	Hot,Cold	1	0.33	0.62
			DF	LRT	Pr(Chi)
Panamá Este	Environment	Per,For	1	5.17	<0.023*
	Month	Jan,Feb,Mar,Aug,Sep	4	19.36	<0.0006*
	SST	Hot,Cold	1	6.11	<0.01*

SST= Sea Surface Temperature, Per= Peridomicile, For= Forest

*Statistically significant (P<0.05)

Table S10 Factors associated with *Lutzomyia panamensis* abundance in Bocas del Toro. For the analysis, we employed a log transformed linear mixed effects model (LMM). In the table the p-values are based on a Chi square test.

Facotors	Estimate	Std. Error	P
Intercept	1.93	0.89	<0.05*
Environment	1.00	1.10	0.28
Quarter Fourth	3.48	1.01	0.03905*
Quarter Second	1.22	1.05	0.03905*
Quarter Third	0.86	1.27	0.03905*
SST Hot	2.68	0.84	0.06
SST Normal	4.26	1.20	0.06
Variance	0.83		
Variance Error	0.91		

SST = Sea Surface Temperature

*Statistically significant (P<0.05)

Table S11 Factors associated with *Lutzomyia gomezi* abundance in Coclé. For the analysis, we employed a log transformed linear mixed effects model (LMM). In the table, the p-values are based on a Chi square test.

Facotors	Estimate	Std. Error	P
Intercept Perdomicile	2.48	0.40	<0.05*
Environment	2.57	0.52	0.83
Month Jan.	0.77	0.32	<0.0001*
Month Jul.	-0.18	0.70	<0.0001*
Month Mar.	0.33	0.64	<0.0001*
SST Hot	4.21	0.50	0.0002534*
SST Normal	2.94	0.68	0.0002534*
Variance	0.39		
Variance Error	0.39		

SST = Sea Surface Temperature

*Statistically significant ($P<0.05$)

Table S12 Factors associated with *Lutzomyia panamensis* abundance in Coclé. For the analysis, we employed a log transformed linear mixed effects model (LMM). In the table, the p-values are based on a Chi square test.

Facotors	Estimate	Std. Error	P
Intercept	3.62	0.64	<0.05
Environment Perdomicile	3.36	0.88	0.74
Month Jan.	1.39	0.45	3.922e-4*
Month Jul.	1.78	0.98	3.922e-4*
Month Mar.	1.36	0.92	3.922e-4*
SST Hot	5.00	0.72	0.0348712*
Variance	1.12		
Variance Error	0.77		

SST = Sea Surface Temperature

*Statistically significant (P<0.05)

Table S13 Factors associated with *Lutzomyia trapidoi* abundance in Coclé. For the analysis, we employed a log transformed linear mixed effects model (LMM). In the table, the p-values are based on a Chi square test.

Factors	Estimate	Std. Error	P
Intercept	2.49	0.44	<0.05
Environment Perdomicile	1.71	0.50	0.09
Month Jan.	0.66	0.45	0.000457*
Month Jul.	-1.18	0.99	0.000457*
Month Mar.	-0.62	0.90	0.000457*
SST Hot	4.93	0.71	0.001411*
SST Normal	4.11	0.99	0.001411*
Variance	0.25		
Variance Error	0.79		

SST = Sea Surface Temperature

*Statistically significant (P<0.05)

Table S14 Factors associated with *Lutzomyia gomezi* abundance in Colón. For the analysis, we employed a log transformed a linear mixed effects model (LMM). In the table, the p-values are based on a Chi square test.

Facotors	Estimate	Std. Error	P
Intercept	0.06	0.46	>0.05
Environment Perdomicile	0.88	0.37	0.06
Month Aug.	0.09	0.82	0.04292*
Month Feb.	1.02	0.65	0.04292*
Month Jan.	-0.18	0.66	0.04292*
Month Mar.	0.21	0.59	0.04292*
Month May	-1.34	0.60	0.04292*
Month Nov	2.71	0.95	0.04292*
Month Oct.	0.66	0.46	0.04292*
Month Sep.	-0.40	0.76	0.04292*
SST Hot	1.08	0.47	0.01068*
SST Normal	1.17	0.76	0.01068*
Variance	0.20		
Variance Error	0.67		

SST = Sea Surface Temperature

*Statistically significant (P<0.05)

Table S15 Factors associated with *Lutzomya panamensis* abundance in Colón. For the analysis, we employed a log transformed linear mixed effects model (LMM). In the table, the p-values are based on a Chi square test.

Facotors	Estimate	Std. Error	P
Intercept	2.20	0.40	<0.05
Environment Peridomicile	0.82	0.42	0.004569*
Quarter Fourth	3.45	0.42	<0.0001*
Quarter Second	0.30	0.53	<0.0001*
Quarter Third	2.18	0.28	<0.0001*
SST Hot	2.94	0.39	<0.0001*
SST Normal	5.59	0.58	<0.0001*
Variance	0.35		
Variance Error	0.51		

SST = Sea Surface Temperature

*Statistically significant (P<0.05)

Table S16 Factors associated with *Lutzomyia trapidoi* abundance in Colón. For the analysis, we employed log a transformed linear mixed effects model (LMM). In the table, the p-values are based on a Chi square test.

Facotors	Estimate	Std. Error	P
Intercept	0.33	0.49	>0.05
Environment Perdomicile	-0.49	0.43	0.05
Month Aug.	0.70	0.79	0.0139737*
Month Feb.	1.24	0.66	0.0139737*
Month Jan.	1.51	0.70	0.0139737*
Month Mar.	1.47	0.63	0.0139737*
Month May	-0.08	0.53	0.0139737*
Month Nov.	4.04	0.97	0.0139737*
Month Oct.	1.23	0.49	0.0139737*
Month Sep.	1.17	0.76	0.0139737*
SST Hot	0.96	0.47	0.0009515*
SST Normal	2.44	0.76	0.0009515*
Variance	0.33		
Variance Error	0.67		

SST = Sea Surface Temperature

*Statistically significant (P<0.05)

Table S17 Factors associated with *Lutzomyia gomezi* abundance in Darién. For the analysis, we employed a log transformed linear mixed effects model (LMM). In the table, the p-values are based on a Chi square test.

Facotors	Estimate	Std. Error	P
Intercept	0.3758	0.3236	>0.05
Environment Perdomicile	0.47	0.33	0.30
Season Rainy	-1.33	0.36	<0.0001*
SST Hot	-2.28	0.36	<0.0001*
SST Normal	-1.77	0.31	<0.0001*
Variance	0.08		
Variance Error	0.38		

SST = Sea Surface Temperature

*Statistically significant (P<0.05)

Table S18 Factors associated with *Lutzomyia gomezi* abundance in Panamá Este. For the analysis, we employed a log transformed linear model (LM). In the table the p-values are based on a t-test.

Facotors	Estimate	Std. Error	t-value	Pr(>|t|)
Intercept	-1.35	1.05	-1.29	0.22
Environment Perdomicile	-0.85	0.73	0.68	0.51
Month Feb.	0.01	0.90	1.51	0.15
Month. Jan.	1.60	1.24	2.38	0.02838 *
Month Mar.	-0.26	1.10	0.99	0.34
Month Sep.	1.31	1.11	2.39	0.02807 *
SST Hot	1.23	0.83	3.13	0.00584 *
SST Normal	-0.50	1.52	0.56	0.58
Multiple R-squared	1.13			

SST = Sea Surface Temperature

*Statistically significant (P<0.05)

Table S19 Factors associated with *Lutzomyia panamensis* abundance in Panamá Este. For the analysis, we employed a log transformed linear mixed effects model (LMM). In the table, the p-values are based on a Chi square test.

Facotors	Estimate	Std. Error	P
Intercept	0.04	0.63	>0.05
Environment Peridomicile	-0.82	0.45	0.023014*
Month Feb.	0.88	0.49	0.000667*
Month Jan.	1.18	0.64	0.000667*
Month Mar.	0.20	0.61	0.000667*
Month Sep.	2.57	0.65	0.000667*
SST Hot	0.94	0.43	0.013446*
Variance	0.38		
Variance Error	0.34		

SST = Sea Surface Temperature

*Statistically significant ($P<0.05$)

Table S20 Factors associated with *Lutzomyia trapidoi* abundance in Panamá Este. For the analysis, we employed a log transformed linear mixed effects model (LMM). In the table, the p-values are based on a Chi square test.

Facotors	Estimate	Std. Error	P
Intercept	-0.25	0.89	>0.05
Environment Perdomicile	-0.62	0.64	0.49
Month Feb.	1.00	0.72	0.12
Month Jan.	1.34	0.94	0.12
Month Mar.	0.09	0.89	0.12
Month Sep.	1.04	0.90	0.12
SST Hot	0.92	0.63	0.01946*
SST Normal	2.89	1.34	0.01946*
Variance	0.52		
Variance Error	0.74		

SST = Sea Surface Temperature

*Statistically significant (P<0.05)

Table S21 Factors associated with *Lutzomya gomezi* abundance in Panamá Oeste. For the analysis, we employed a log transformed linear mixed effects model (LMM). In the table, the p-values are based on a Chi square test.

Facotors	Estimate	Std. Error	P
Intercept	1.80	0.35	<0.05*
Environment Forest	1.11	0.33	0.07
Environment Peridomicile	1.25	0.29	0.07
Quarter Fourth	1.65	0.22	0.009501*
Quarter Second	1.31	0.36	0.009501*
Quarter Third	1.10	0.22	0.009501*
SST Hot	2.43	0.26	0.007886*
SST Normal	2.62	0.35	0.007886*
Variance	0.40		
Variance Error	0.70		

SST = Sea Surface Temperature

*Statistically significant (P<0.05)

Table S22 Factors associated with *Lutzomyia panamensis* abundance in Panamá Oeste. For the analysis, we employed a log transformed linear mixed effects model (LMM). In the table, the p-values are based on a Chi square test.

Facotors	Estimate	Std. Error	P
Intercept	-0.84	0.70	>0.05
Environment Forest	0.06	0.41	0.10
Environment Peridomicile	-0.28	0.37	0.10
Month Aug.	2.53	1.29	<0.0001*
Month Dec.	1.39	0.83	<0.0001*
Month Feb.	1.75	0.63	<0.0001*
Month Jan.	0.81	0.64	<0.0001*
Month Jul.	1.06	0.67	<0.0001*
Month Jun.	-0.39	0.80	<0.0001*
Month Mar.	-0.03	0.66	<0.0001*
Month May	-0.94	0.71	<0.0001*
Month Nov.	2.07	0.63	<0.0001*
Month Oct.	1.73	0.60	<0.0001*
Month Sep.	1.37	0.63	<0.0001*
SST Hot	-0.39	0.42	0.003923*
SST Normal	1.08	0.60	0.003923*
Variance	0.45		
Variance Error	1.32		

SST = Sea Surface Temperature

*Statistically significant (P<0.05)

Table S23 Factors associated with *Lutzomyia trapidoi* abundance in Panamá Oeste. For the analysis, we employed a log transformed linear mixed effects model (LMM). In the table, the p-values are based on a Chi square test.

Facotors	Estimate	Std. Error	P
Intercept	0.26	0.54	>0.05
Environment Forest	-0.06	0.34	0.43
Environment Perdomicile	-0.07	0.29	0.43
Month Aug.	0.10	0.96	<0.0001*
Month Dec.	2.61	0.61	<0.0001*
Month Feb.	1.97	0.45	<0.0001*
Month Jan.	2.08	0.47	<0.0001*
Month Jul.	0.87	0.49	<0.0001*
Month Jun.	0.74	0.52	<0.0001*
Month Mar.	0.88	0.47	<0.0001*
Month May	-0.28	0.37	<0.0001*
Month Nov.	1.97	0.49	<0.0001*
Month Oct.	1.69	0.45	<0.0001*
Month Sep.	1.26	0.46	<0.0001*
SST Hot	0.20	0.33	0.46
SST Normal	0.74	0.43	0.46
Variance	0.54		
Variance Error	0.78		

SST = Sea Surface Temperature

*Statistically significant (P<0.05)

Table S24 Factors associated with *Lutzomyia gomezi* abundance in Veraguas. For the analysis, we employed a linear model (LM). In the table the p-values are based on a t-test.

Facotors	Estimate	Std. Error	t-value	Pr(>|t|)
Intercept	1.50	0.63	2.37	0.0339 *
Season Rainy	0.12	0.61	-2.26	0.0420 *
SST Hot	2.08	0.34	1.68	0.12
Multiple R-squared	0.52			

SST = Sea Surface Temperature

*Statistically significant (P<0.05)

Table S25 Factors associated with *Lutzomyia panamensis* abundance in Veraguas. For the analysis, we employed a log transformed linear mixed effects model (LMM). In the table, the p-values are based on a Chi square test.

Facotors	Estimate	Std. Error	P
Intercept	0.00	0.64	>0.05
Quarter Fourth	1.29	0.74	0.03594*
Quarter Second	0.27	0.71	0.03594*
Variance	0.12		
Variance Error	0.29		

SST = Sea Surface Temperature

*Statistically significant (P<0.05)
